# DNA methylation biomarker analysis from low-survival-rate cancers based on genetic functional approaches

**DOI:** 10.3389/fbinf.2025.1523524

**Published:** 2025-01-28

**Authors:** Yi-Hsuan Tsai, Prasenjit Mitra, David Taniar, Tun-Wen Pai

**Affiliations:** ^1^ Department of Computer Science and Information Engineering, National Taipei University of Technology, Taipei, Taiwan; ^2^ College of Information Science and Engineering, The Pennsylvania State University, University Park, PA, United States; ^3^ Department of Software Systems and Cybersecurity, Monash University, Clayton, VIC, Australia; ^4^ Department of Computer Science and Engineering, National Taiwan Ocean University, Keelung, Taiwan

**Keywords:** comorbidity pattern, support vector machine, early detection, KEGG pathway, gene ontology

## Abstract

Identifying cancer biomarkers through DNA methylation analysis is an efficient approach toward the detection of aberrant changes in epigenetic regulation associated with early-stage cancer types. Among all cancer types, cancers with relatively low five-year survival rates and high incidence rates were pancreatic (10%), esophageal (20%), liver (20%), lung (21%), and brain (27%) cancers. This study integrated genome-wide DNA methylation profiles and comorbidity patterns to identify the common biomarkers with multi-functional analytics across the aforementioned five cancer types. In addition, gene ontology was used to categorize the biomarkers into several functional groups and establish the relationships between gene functions and cancers. ALX3, HOXD8, IRX1, HOXA9, HRH1, PTPRN2, TRIM58, and NPTX2 were identified as important methylation biomarkers for the five cancers characterized by low five-year survival rates. To extend the applicability of these biomarkers, their annotated genetic functions were explored through GO and KEGG pathway analyses. The combination of ALX3, NPTX2, and TRIM58 was selected from distinct functional groups. An accuracy prediction of 93.3% could be achieved by validating the ten most common cancers, including the initial five low-survival-rate cancer types.

## 1 Introduction

Cancers are highly complex diseases, and no ideal prophylactic, diagnostic, or therapeutic methods are currently available for them. Although we can reduce cancer risk by avoiding some important preventable risk factors, such as not smoking, not using alcohol and maintaining a healthy weight, there is no guarantee that someone will not develop cancers. Certain cancers may be asymptomatic in the early stages, and by the time patients do present with symptoms, the diseases might have already progressed to the more advanced stages when the cancers have metastasized (Beger et al., 2008). By that time, treatment may become very difficult, and the survival rate is low. The five-year survival rates for pancreatic, esophageal, liver, lung, and brain cancers are all less than 30% compared to other cancers (Siegel et al., 2021; Deorah et al., 2006). This study focused on analyzing genome-wide DNA methylation profiles simultaneously for these five foregoing cancer types, as they all have high incidence and low survival. Highly associated methylation biomarkers were identified simultaneously from different cancers on a genome-wide scale, which could be applied to detect whether a subject possesses a higher risk of developing the selected target cancers.

Common causes of cancer include genetic abnormalities, structural variations, and abnormal gene expression resulting from DNA methylation (Mardis and Wilson, 2009). In the present study, we selected biomarkers for early cancer diagnosis based on DNA methylation mechanisms. DNA methylation regulates gene expression without altering DNA sequences. Hence, DNA methylation is a type of epigenetics. Unlike true genetics, epigenetics focuses on the changes in gene function that occur in response to environmental factors, histone modifications, chromatin conformation, and noncoding RNAs (Zhang et al., 2020; Frías-Lasserre and Villagra, 2017).

In regular DNA methylation, CH_3_ is attached to C-5 of cytosine by DNA methyltransferases, and 5-methylcytosine is formed (Moore et al., 2013). Gene expression decreases with an increasing degree of DNA methylation. In mammals, DNA methylation usually occurs at CpG sites where a guanine nucleotide follows a cytosine nucleotide and they are linked by a phosphate moiety. The C + G content and the observed: expected CpG ratio of a CpG-rich CpG island are >50% and >0.6, respectively (Gardiner-Garden and Frommer, 1987). Cancer risk increases with tumor suppressor gene methylation and oncogene demethylation. Methylated and unmethylated probes occur at methylation sites, and their methylation levels are indicated as β-values. The latter are obtained by dividing the signal intensity of the methylated probe by the signal intensity of all probes with normalized values between 0 and 1 (Du et al., 2010). Here, we identified highly discriminating biomarkers by determining the differences in methylation between tumor and normal cells at each probe.

Earlier studies performed differential gene expression (RNA-seq and DNA methylation) analyses and performed gene functional clustering and pathway analyses to obtain biomarkers related to specific diseases (Sun et al., 2021; Yang et al., 2019). In the present work, we combined the output of DNA methylation analyses and comorbidity patterns for specific target cancers. We then identified superior candidate biomarkers by intersecting primary biomarkers identified by the DNA methylation profile analysis with the secondary biomarkers related to the comorbidities of each specific cancer type. The most recent research has obtained biomarkers for specific cancers by profiling DNA methylation analyses either on single cancers or those within similar organ systems. However, these biomarkers might also be common to other cancer types and could misidentify or erroneously detect them. The aims of this study were to find commonly associated biomarkers for the foregoing five cancers and extend to other cancer types, and try to develop a better and effective diagnostic tool for general cancer detection at early stages.

## 2 Materials and methods

### 2.1 Differential methylation analysis for the primary biomarkers

The Cancer Genome Atlas (TCGA; https://www.genome.gov/Funded-Programs-Projects/Cancer-Genome-Atlas) was the source of the DNA methylation profiles for >50 cancer types acquired from the Infinium HumanMethylation450 K BeadChip (Illumina, San Diego, CA, United States). Each profile included the methylation levels (β-value) for approximately 480,000 probes. Tumor tissue samples were assigned to the experimental group, while normal tissue samples were assigned to the control group. The numbers of subjects per group, cancer type, and tumor type are listed in Table 1. For the TCGA datasets, we listed the Sentrix ID and Sentrix position of each subject, which match the corresponding IDAT file in Supplementary Table S1.

**TABLE 1:** The numbers of patients per group, cancer type, and tumor type.

Cancer	Source	Number of the subjects in experimental group	Number of the subjects in control group	Tumor type	Accession number
Brain	GEO	70	8	Glioma	GSE123678
Esophagus	TCGA	186	16	Esophageal Carcinoma	N/A
Liver	TCGA	280	36	Liver Hepatocellular Carcinoma	N/A
Lung	TCGA	370	42	Lung Squamous Cell Carcinoma	N/A
Pancreas	TCGA	185	10	Pancreatic Adenocarcinoma	N/A

In accordance with standard DNA methylation analytical procedures, the IDAT file required standard preprocessing, such as data quality control (QC) and β-value normalization (Wang et al., 2018). Here, the Chip Analysis Methylation Pipeline (ChAMP) toolkit (Morris et al., 2014) was used to evaluate the methylation profiles. Probes unsuitable for analysis were removed by QC procedures. BMIQ normalization procedures were applied to correct the scale differences introduced by the probe design (Teschendorff et al., 2013). As the β-values for certain probes may not be distributed within the majority ranges because of noise interference, the interquartile range method (Walfish, 2006) was applied to remove outliers for each probe. The Benjamini‒Hochberg multiple-testing correction (Benjamini and Hochberg, 1995) was applied to the *p* values to lower the false discovery rate (FDR) and to filter the probes. The data were preprocessed and cleaned, and the average beta-value difference (*∆β* value) between the experimental and control groups was calculated for each probe. If a gene contained at least one probe (loci) with |∆β| value greater than a previously defined thresholding value and its p-value was less than 0.05, it would be considered as a primary biomarker for the target cancer. The workflow of our analyses was step-by-step shown in the Supplementary Figure S1.

### 2.2 Comorbidity pattern analysis for the secondary biomarkers

Certain diseases may occur before and/or after a cancer is diagnosed. These comorbidities have certain associations with cancers and could play vital roles in cancer prevention, diagnosis, prognosis, and treatment (Ogle et al., 2000). Therefore, the biomarkers were selected by considering the characteristics of the comorbidities related to a specific cancer type. Relevant studies and reports on a selected cancer and its comorbidities were searched, and the associated genes could be identified from the DisGeNet (https://www.disgenet.org) and OMIM (https://www.omim.org) databases. The comorbidities and their associated genetic biomarkers for each cancer type were defined as secondary biomarkers.

### 2.3 Common biomarker selection

Testing toolkit costs must be considered when methylation-specific PCR assays are performed for early cancer detection. Hence, the number of target methylation biomarkers should be reduced to a reasonable figure. We expected that the number of target biomarkers could be reduced as much as possible, and that higher classification performance could be achieved. Methylation biomarkers with significantly different performance levels among the five cancers had to be carefully selected to evaluate the DNA methylation status of the query subjects. The results of the initial screening indicated whether additional experimentation or examination is needed.

For common biomarker selection, a threshold of |*Δβ|* values >0.2 was applied to all five selected cancers simultaneously. The biomarkers that met this condition possessed high differential methylation expression levels across all five selected cancers. These biomarkers were hierarchically clustered (Chen et al., 2014) into different functional groups, and only one representative biomarker was selected from each functional group.

### 2.4 Gene distance calculation and functional clustering

Each gene might be associated with multiple functions and annotated by several well-known functional annotation databases. Hence, functional relationships among all selected biomarker candidates should be analyzed, and representative biomarkers can be assigned based on their functionality. Here, gene ontology (GO) annotations (geneontology.org) were used to cluster the genes according to their annotated functional terms among three GO trees. The associated GO terms were arranged by a directed acyclic graph (DAG) tree structure (Bada et al., 2004). When the GO terms associated with the biomarker genes and their precise locations in the tree structures were identified, the distances between gene pairs could be measured, and a distance matrix of all candidate biomarkers was generated.

The weight of a specific GO term is defined before calculating gene distances, and it is calculated by counting the number of genes annotated by the *i*th GO term (*G*_*ti*_) divided by the total number of nonduplicate genes within all GO terms. The weight of a GO term is used as a reference for the position located in a specific GO tree. The GO terms located in the upper levels of a GO tree contain relatively more annotated genes, and their weights are relatively higher. Equation 1 shows the calculation formula for an associated weight. *W* (*t*_*i*_) represents the weight of the *i*th GO term. The information content and Sorensen-Dice coefficient distances (Sorensen, 1948) were then applied to calculate the gene distances. If two GO terms of interest were located in different GO functional trees, they would have no common ancestor, and their information content distance would be 1. However, if two GO terms were located in the same GO tree, they might have at least one or more common ancestors. In this case, the weight of the lowest common ancestor (LCA) was calculated according to the information content distance (*dist*_IC_) and denoted in Equation 2. Here, *t*_*LCAi*,*j*_ is the LCA for the *t*_*i*_ and *t*_*j*_ GO terms. The Sorensen-Dice coefficient distance (*dist*_SC_) is a statistical method used to determine the similarity between two sets. It was applied to identify similarities between the gene sets annotated by GO terms. If *G*_*ti*_ and *G*_*tj*_ are gene sets annotated by the *i*th and *j*th GO terms individually, then the Sorensen-Dice coefficient distance is calculated according to Equation 3. Here, *G*_*ti*_∆*G*_*tj*_ is the symmetric difference between *G*_*ti*_ and *G*_*tj*_. The distance between two GO terms may be measured by calculating the average information content and Sorensen-Dice coefficient distances (shown in Equation 4). The functional distance between genes *a* and *b* is determined by averaging the distances between GO term pairs for *a* and *b*. Once all distances for candidate biomarker pairs are calculated, a distance matrix can be formulated and normalized between 0 and 1. If the functional relationship between two genes is close, their distance would be close to 0. If two genes are not annotated by common GO terms, their distance would be 1. After the distance matrix was constructed, the following clustering analysis was performed for all selected candidate biomarkers.

Algorithms were used to cluster candidate biomarkers into several functional groups according to the measured distance matrix of gene functions. Genes with similar functions were classified into the same group. Both partitioning and hierarchical clustering algorithms were applied in this study. However, the hierarchical clustering approach is more suitable for categorical data as long as a similarity measure can be defined accordingly, and no specific number of final biomarkers is defined at the beginning. Hence, the hierarchical clustering approach is a preferable choice.

Furthermore, KEGG pathway analysis was also performed for each selected cancer by using the GSEA package in Python (GSEAPY) (Fang et al., 2023). This analysis yielded the shared KEGG pathways among the five selected cancers. For each selected cancer, biomarkers with |Δ*β*| greater than 0.2 were utilized to form an input gene set for KEGG pathway analysis. After that, we performed an intersection of KEGG pathways for each cancer, and the intersected genes within the same pathway in each cancer were specifically selected.

$$W\left(t_{i}\right)=\frac{\left|G_{t_{i}}\right|}{\#\;\text{of}\;\text{non}-\text{duplicate}\;\text{genes}}$$ (1)

$${dist}_{\text{IC}}\left(t_{i},t_{j}\right)=\left\{\begin{array}{l} 2W\left(t_{{LCA}_{i,j}}\right)-W\left(t_{i}\right)-W\left(t_{j}\right),t_{LCA}\text{ exists }\\ 1,\text{otherwise} \end{array}\right.$$ (2)

$${dist}_{\text{SC}}\left(t_{i},t_{j}\right)=\frac{\left|G_{t_{i}}∆G_{t_{j}}\right|}{\left|G_{t_{i}}\cup G_{t_{j}}\right|+\left|G_{t_{i}}\cap G_{t_{j}}\right|}$$ (3)

$$dist\left(t_{i},t_{j}\right)=\frac{{dist}_{\text{IC}}\left(t_{i},t_{j}\right)+{dist}_{\text{SC}}\left(t_{i},t_{j}\right)}{2}$$ (4)

### 2.5 Identifying the optimal biomarker combination

To find the biomarker combination with the best performance, the selected common biomarker candidates were isolated individually or arranged into multiple groups, and *β*-values were obtained for each subject. Support Vector Machine (SVM) was applied to select the optimal biomarker combination based on the classification accuracy of each biomarker group (Boser et al., 1992). The training cohort for the SVM comprised the subjects diagnosed with five low-survival-rate cancers. To evaluate the performance of each biomarker combination, we integrated testing datasets obtained from Gene Expression Omnibus (GEO; https://www.ncbi.nlm.nih.gov/geo/). GEO is a database repository containing comprehensive genetic and epigenetic datasets as independent validation resources for selected biomarker evaluation (Bjaanæs et al., 2016; Soares-Lima et al., 2021; Nones et al., 2014). The numbers of testing subjects per testing dataset are listed in Table 2. To ensure the commonality of each common biomarker, the methylation profiles of subjects diagnosed with the most prevalent cancers (breast, colorectal, prostate, bladder, and stomach) were additionally included from TCGA into the testing cohort. Hence, a total of 10 cancer types were applied to test the performance of each biomarker combination selected from the 8 common biomarkers, and the optimal biomarker combination was selected based on an overall testing accuracy and functionally clustered groups.

**TABLE 2:** The numbers of subjects per group, cancer type, and tumor type. (Test cohort).

Cancer	Source	Number of the subjects in experimental group	Number of the subjects in control group	Tumor type	Accession number
Brain	TCGA	534	0	Brain Lower Grade Glioma	N/A
Esophagus	GEO	24	16	Esophageal squamous cell carcinoma	GSE178212
Liver	TCGA	100	14	Liver Hepatocellular Carcinoma	N/A
Lung	GEO	164	19	Lung adenocarcinomas	GSE66836
Pancreas	GEO	167	29	Pancreatic ductal adenocarcinoma	GSE49149
Colorectal	TCGA	314	38	Adenomas and adenocarcinomas	N/A
Breast	TCGA	368	47	Ductal and lobular neoplasms	N/A
Bladder	TCGA	419	21	Transitional cell papillomas and carcinomas	N/A
Prostate	TCGA	503	50	Adenomas and adenocarcinomas	N/A
Stomach	TCGA	395	2	Adenomas and adenocarcinomas	N/A

To verify the applicability of the optimal biomarker combination for individual cancer types, we performed two additional tests. Firstly, we applied SVM to independently train and test for each of the selected low-survival-rate cancers. Next, we combined the subjects from the five selected low-survival-rate cancers to train a universal prediction model based on SVM technology, and the prediction model was evaluated on the five additional selected cancers (breast, colorectal, prostate, bladder, and stomach) to validate the classification performance. The numbers of subjects for different groups, cancer types, and tumor types were listed on the Table 2.

## 3 Results

### 3.1 Primary biomarkers

**FIGURE 1:**
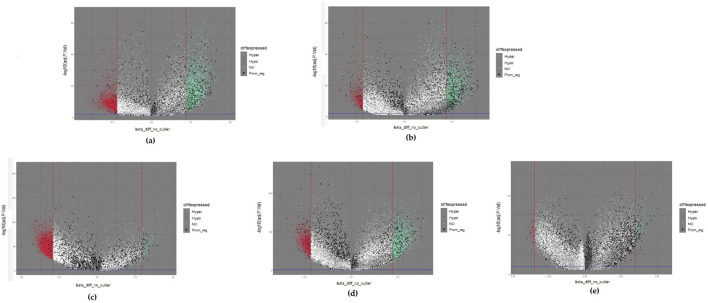
Volcano plots of five selected cancers: **(A)** colorectal cancer; **(B)** esophageal cancer; **(C)** liver cancer; **(D)** lung cancer; **(E)** pancreatic cancer. Hypermethylated methylation loci (Hyper) were represented by light green dots, and hypomethylated methylation loci (Hypo) were represented by light red dots. The black dots represented the loci near the promoter region (Prom_reg).

Differentially methylated positions (DMPs) were obtained by setting the thresholds |∆β values| ≥ 0.35 and Benjamini-Hochberg adjusted *p*-values <0.01. We obtained 8,724, 4,337, 7,607, 4,765, and 452 DMPs for brain, esophageal, liver, lung, and pancreatic cancer, respectively. We then used volcano plots to show the distribution of all DMPs (Figure 1). The horizontal axis indicates ∆β values. The DMPs approaching both sides outwardly reflect large differences in methylation. The vertical axis reveals that the statistical significance of the DMPs increases with decreasing p value. Therefore, the DMPs located at the upper right and upper left corners of the volcano plot are good candidates. We also color-coded the DMPs in the volcano plot based on their methylation status. If a DMP |∆β| value is larger than the thresholding value, the DMP was hypermethylated and represented by a light green dot. If a DMP |∆β| value is less than the thresholding value, the DMP was hypomethylated and represented by a red dot. If the DMPs were located within promoter regions, they probably regulated gene expression (Li and Zhang, 2014) and served as good biomarker candidates for the following experimental design. These DMPs are represented by black dots. The remaining DMPs are represented by white dots. After DMPs were filtered by the defined |∆β| values threshold, 3,227, 1,342, 1,615, 1,383, and 240 genes remained for brain, esophageal, liver, lung, and pancreatic cancer, respectively. These DMPs were defined as the primary biomarkers.

### 3.2 Comorbidities and secondary biomarkers

The comorbidities associated with each cancer were retrieved from published articles. Their associated genes were identified from well-known gene-disease databases. For example, the comorbidities of brain cancer are related to benign brain and nervous system neoplasms. Esophageal cancer comorbidities are related to certain bone pathologies. Melo-Martin et al. reported that a lack of aldehyde dehydrogenase 2 (ALDH2) may cause Asian alcohol flush syndrome, which is correlated with esophageal cancer and osteoporosis (de Melo-Martin and Crystal, 2021). Elliott et al. stated that patients with esophageal cancer are at increased risk of osteoporosis even after esophagectomy (Elliott et al., 2019). Liver cancer comorbidities are associated with cirrhosis and hepatitis B and C. Tatsuo Kanda et al. indicated that most patients with hepatocellular carcinoma (HCC) also have cirrhosis, and ∼70% of all patients with HCC also have hepatitis B or C (Kanda et al., 2019). The most common lung cancer comorbidities include pneumonia and airway-related diseases. Alessia Guarnera et al. reported that COVID-19 pneumonia may affect lung cancer diagnosis (Guarnera et al., 2022). Patients with lung cancer are relatively more susceptible to COVID-19 pneumonia than noncancerous patients. There were 20,376, 1,203, 4,065, 962, and 12,291 associated disease genes (secondary biomarkers) associated with brain, esophageal, liver, lung, and pancreatic cancer, respectively. Information and references for the comorbidities are shown in Table 3.

**TABLE 3:** Information and references for comorbidities of five cancers.

ICD-single	Cancer	Comorbidities	GeneNum	References
225	Brain	Benign neoplasms of brain and other parts of nervous system	10170	Sturm et al. (2017)
239	Brain	Neoplasms of unspecified nature	10206	Sturm et al. (2017)
733	Esophagus	Other disorders of bone and cartilage	1203	Elliott et al. (2019)
571	Liver	Chronic liver disease and cirrhosis	649	Roca Suarez et al. (2021), Kanda et al. (2019)
070	Liver	Viral hepatitis	1780	Ringehan et al. (2017)
574	Liver	Cholelithiasis	269	Wang et al. (2019)
573	Liver	Other disorders of liver	1367	Wang et al. (2019)
486	Lung	Pneumonia	216	Guarnera et al. (2022), Søyseth et al. (2007)
496	Lung	Chronic airway obstruction	208	Caplin and Festenstein (1975)
491	Lung	Chronic bronchitis	299	Caplin and Festenstein (1975)
490	Lung	Bronchitis	239	Caplin and Festenstein (1975)
577	Pancreas	Diseases of pancreas	763	Umans et al. (2021)
574	Pancreas	Cholelithiasis	269	Monami et al. (2017)
532	Pancreas	Duodenal ulcer	120	Higashiyama et al. (2015)
571	Pancreas	Chronic liver disease and cirrhosis	649	Xu et al. (2013), Gdowski et al. (2017)
211	Pancreas	Benign neoplasms of other parts of digestive system	10169	Basturk and Askan (2016)
533	Pancreas	Peptic ulcer site unspecified	168	Bao et al. (2010)
531	Pancreas	Gastric ulcer	153	Bao et al. (2010)

### 3.3 KEGG pathway analysis of each selected cancer

We applied the GSEA package for discovering shared significant KEGG pathways among the five selected cancers. This analytical procedure identified 141 common KEGG pathways with an adjusted p-value below 0.05. The name of each pathway and their corresponding intersected genes were listed in Supplementary Table S2.

### 3.4 Functional clustering and KEGG pathways of common biomarkers

**FIGURE 2:**
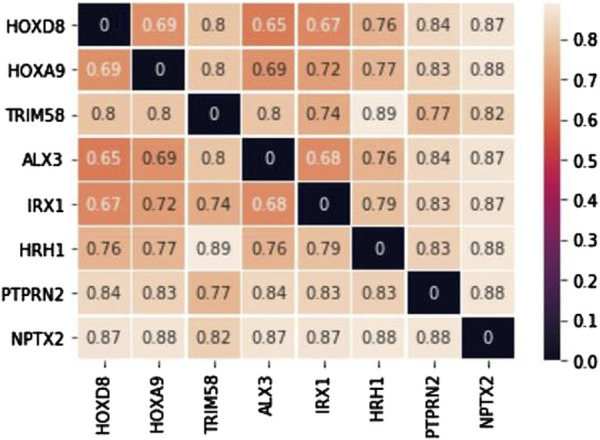
The distance matrix for 8 common biomarkers.

The candidate biomarkers were obtained by intersecting the primary and secondary biomarkers, which have characteristics of both methylation and comorbidity patterns. The numbers of candidate biomarkers for brain, esophageal, liver, lung, and pancreatic cancers are 1,692, 725, 716, 773, and 156, respectively. We then selected the biomarkers from each selected cancer that met the conditions of a ∆β values greater than 0.2 to form five biomarker sets, and their intersection was defined as common biomarkers. Finally, there were eight biomarkers could be identified including ALX3, HOXA9, HOXD8, HRH1, IRX1, NPTX2, PTPRN2, and TRIM58. Among them, only HRH1 and PTPRN2 were hypomethylated, while the other six common biomarkers were hypermethylated conditions. After gene distance calculation and distance matrix construction (Figure 2) for the eight aforementioned consensus biomarkers, we used the unweighted pair group method with arithmetic mean (UPGMA) to divide them into three groups. The first group comprised ALX3, HOXD8, IRX1, HOXA9, and HRH1, the second group included PTPRN2 and TRIM58, and the third group contained the last biomarker of NPTX2. For the first functional group, ALX3, HOXD8, IRX1, and HOXA9 shared GO terms in all three GO categories. The common GO terms were regulation of transcription from the RNA polymerase II promoter under the GO structural tree of Biological_Processes, chromatin under the GO structural tree of Cellular_Component, and sequence-specific double-stranded DNA binding under the GO structural tree of Molecular_Function. In addition to GO functional analysis, the associated KEGG pathways were also found as follows: HOXA9 was located in hsa05202 (Transcriptional misregulation in cancer), HRH1 in hsa04020 (Calcium signaling pathway), hsa04080 (Neuroactive ligand-receptor interaction), and hsa04750 (Inflammatory mediator regulation of TRP channels). Both Zhang and Yin mentioned that the pathway hsa05202 was related to non-small cell lung cancer and hepatocellular carcinoma, respectively (Zhang et al., 2019; Yuan et al., 2021). Xu et al. revealed that the apoptosis of lung cancer cells is induced through calcium signaling pathway (Xu et al., 2015).

### 3.5 The optimal biomarker combination

We further compared the performances of the eight selected biomarker candidates to evaluate various combinations and different numbers of biomarkers for the prediction of five low five-year survival rate cancer types (brain, esophageal, liver, lung, and pancreatic cancers). In this study, we also selected additional five common cancer types (breast, colorectal, prostate, bladder, and stomach cancers) to validate the commonality of the selected cancer biomarkers. Considering the diversity of genetic functions, one biomarker from each functional group clustered based on the GO functional annotations was selected to form a biomarker combination. We found that the biomarker combination with the highest classification accuracy consisted of ALX3, NPTX2, and TRIM58, which could achieve an average accuracy of 93.3% for the original five low five-year survival rate cancers and the other five additional common cancers (breast, colorectal, prostate, bladder, and stomach cancers). The recall and precision for the 10 different cancer types could achieve an average of 0.957 and 0.97, respectively.

Two additional tests based on the optimal biomarker combination (ALX3, NPTX2, and TRIM58) were performed in this study. The first test executed independent training and testing procedures for the initially selected low-survival-rate cancers (brain, esophageal, liver, lung, and pancreatic cancers), and the second test integrated all subjects from the five initially selected low-survival-rate cancers to construct a universal prediction model and applied the developed prediction model to diagnose the five additional selected new cancers (breast, colorectal, prostate, bladder, and stomach cancers) for validation. The corresponding prediction performance of the two tests by featuring the optimal biomarker combination (ALX3, NPTX2, and TRIM58) were shown in Table 4, 5, respectively. In addition, the Δβ values of ALX3, NPTX2, and TRIM58 were shown in Table 6, and the Δβ values for each stage were shown in Table 7. Although no consistent patterns for the Δβ of ALX3, NPTX2, and TRIM58 were observed across the stages, these three genes were stably hypermethylated in nearly all stages, except for NPTX2 at the fourth stage in liver cancer.

**TABLE 4:** Prediction results of independent prediction models for the five low-survival-rate cancers by using the optimal biomarker combination (ALX3, NPTX2, and TRIM58).

Cancer	Accuracy	Recall	Precision	F score
Brain	0.885	0.871	1.000	0.931
Esophagus	0.867	0.733	1.000	0.846
Liver	0.860	0.840	1.000	0.913
Lung	0.885	0.878	0.993	0.932
Pancreas	0.944	0.952	0.981	0.967

**TABLE 5:** Prediction results of the constructed universal model for validating the five additional cancers by using the optimal biomarker combination (ALX3, NPTX2, and TRIM58).

Cancer	Accuracy	Recall	Precision	F score
Colorectal	0.906	0.965	0.932	0.948
Breast	0.964	0.989	0.968	0.979
Bladder	0.952	0.962	0.988	0.975
Prostate	0.888	0.900	0.976	0.936
Stomach	0.987	0.992	0.995	0.994

**TABLE 6:** The Δβ values of ALX3, NPTX2, and TRIM58 for five low-survival-rate cancers.

Cancer	ALX3 (β_tumor, β_normal)	NPTX2	TRIM58
Brain	0.368 (0.395, 0.027)	0.515 (0.535, 0.020)	0.441 (0.463, 0.022)
Esophagus	0.505 (0.660, 0.155)	0.502 (0.694, 0.192)	0.321 (0.563, 0.242)
Liver	0.361 (0.464, 0.103)	0.279 (0.421, 0.142)	0.306 (0.364, 0.058)
Lung	0.573 (0.626, 0.053)	0.288 (0.383, 0.095)	0.463 (0.497, 0.034)
Pancreas	0.395 (0.431, 0.036)	0.455 (0.558, 0.103)	0.396 (0.431, 0.035)

**TABLE 7:** The Δβ values of ALX3, NPTX2, and TRIM58 for five low-survival-rate cancers by stage.

Cancer	ALX3 (1st stage, 2nd stage, 3rd stage, 4th stage)	NPTX2	TRIM58
Esophagus	0.466, 0.476, 0.545, 0.484	0.536, 0.453, 0.514, 0.517	0.351, 0.304, 0.332, 0.326
Liver	0.358, 0.399, 0.344, 0.310	0.294, 0.304, 0.267, −0.104	0.287, 0.329, 0.298, 0.569
Lung	0.559, 0.585, 0.591, 0.621	0.283, 0.306, 0.257, 0.301	0.465, 0.504, 0.383, 0.522
Pancreas	0.318, 0.409, 0.346, 0.426	0.239, 0.472, 0.447, 0.556	0.266, 0.407, 0.392, 0.480

## 4 Discussion

### 4.1 The methylation status of identified biomarkers and patented biomarkers

The best combination of common methylation biomarkers derived from the five initial cancer types were ALX3, NPTX2, and TRIM58. Among them, NPTX2 and TRIM58 were also identified and appeared in certain patents. Most patented biomarkers in Table 8 possessed significant ∆β values in the DNA methylation analytical results and were considered primary biomarkers for specific cancer types. The average ∆β values of the listed patented biomarkers for brain, esophageal, liver, lung, and pancreatic cancers were 0.38, 0.23, 0.25, 0.44, and 0.28, respectively. However, some of the patented biomarkers did not appear in the final biomarker list, mainly because their ∆β values did not satisfy the minimum threshold setting of a specific cancer or their corresponding classification accuracies were too low. For example, in pancreatic cancer, the |∆β values| of SEPT9 fell below the threshold of the default settings; therefore, it was filtered out from the candidate common biomarkers. Furthermore, the number of selected biomarkers should be limited since methylation-specific PCR (MSP) experiments should be considered regarding their materiality of cost. Hence, strict filtering standards and threshold settings were applied in this study for crucial biomarker selection.

**TABLE 8:** The patent for identifying biomarkers through DNA methylation relative to the five cancers.

Patent ID	Cancer	Related biomarkers
US20140011702A1	Brain	TBX3, FSD1, FNDC3B, DGKI, AGT, FLJ25422, SEPP1, SOX10, MAP3K14, SOX10, ACOT8, KCNMB1, CHI3L2, COG4, FAM49A, GPR85, CCND1, MGC29671, LGALS1, SDPR, GPR128, NET1, SLC26A5, RNASE3, CDKN2B, NUP98, CYP24A1, ACTL6B, KLK10, TRPV4, CX36, ***TRIM58***, GRIP1, PHLDA2, PON1, SLC2A2, TNF, FLJ23657, C1orf176, FLJ32447, HOXA11, LY6K, HMG20B, KHDRBS2, WT1, TFF2, ZNF542, ZSCAN1, ZNF540, HBZ, GPR92, HOXA9, KCNA4, RAC2, CYP1B1, FUT3, GCET2, MEGF10, GRK1, GPX5
CN113403398A	Esophageal	NECAB2, UBXN10, CYFIP2
CN110603329A	Liver	BMPR1A, PSD, ARHGAP25, KLF3, PLAC8, ATXN1
US20190203298A1	Lung	BCAT1, ***TRIM58***, ZNF177, CDO1
EP3430162B1	Pancreas	BMP3, RASSF1A, BNC1, MESTv1, TFPI2, APC, SFRP1, SFRP2, EYA2, ***NPTX2***, SEPT9v2, WNT5a, CDKN2B, ALX4, HIC1, RARB, SST, ESR1, TAC1, BRCA1, CHFR, GSTP1, MGMT, MLH1, NEUROG1, P16, PENK and VIM

The genes identified as our final candidates are presented in bold text.

### 4.2 Effects of outliers on biomarker selection

The distribution of the *β* values of cancer patients influences biomarker selection. If there are too many probe outliers, the ∆β values calculation may return major errors, the number of DMPs may decrease if the assigned ∆β values threshold is not changed, and important biomarkers might be initially excluded. O-6-methylguanine-DNA methyltransferase (MGMT) is a critical brain cancer biomarker (Yousefi et al., 2021). If the outliers had not been removed early in the process, the calculated probe ∆β values would be 0.349. The assigned threshold is 0.35. If the outliers were promptly removed, however, the ∆β values calculated for the MGMT probe would increase to 0.443, and MGMT would become one of another biomarker candidates.

### 4.3 Relationships between common biomarkers and cancers

The consensus biomarkers HOXA9 and HOXD8 belong to the HOX gene family. Previous research indicated that HOX genes were associated with liver, colorectal, and lung carcinogenesis. Furthermore, HOXD8 is a downstream gene of certain miRNAs associated with various cancers through cell proliferation and apoptosis (Wen et al., 2020; Sun et al., 2019; Kanai et al., 2010). Among the probes selected from the optimal biomarker combinations, García-Ortiz et al. indicated that methylation levels in circulating NPTX2 increase in pancreatic cancer (García-Ortiz et al., 2023). Skiriutė et al. observed that NPTX2 is highly methylated in glioblastoma (Skiriutė et al., 2013). For TRIM58, Tao et al. showed that TRIM58 is hypermethylated in hepatitis B virus-related hepatocellular carcinoma (HBHC) (Tao et al., 2011). Qiu et al. mentioned that TRIM58 hypermethylation is correlated with poor disease-free survival after hepatectomy (Qiu et al., 2016). Kajiura et al. disclosed that aberrant TRIM58 inactivation may cause early lung adenocarcinoma carcinogenesis (Kajiura et al., 2017). Sun et al. used RNA-seq and DMP analyses, obtained five biomarkers, including TRIM58, and authors showed that TRIM58 is a hypermethylated biomarker for pancreatic cancer (Sun et al., 2021).

Optimal combinations of the consensus biomarkers for the five cancer types revealed that classification accuracy was relatively low when we only selected one or two biomarkers from a functional group. Moreover, classification accuracy did not differinate or be improved remarkably even when more than 3 biomarkers were selected from the same functional group.

### 4.4 Effects of liquid biopsy methylation profiles on associated KEGG pathway

To obtain tissue biopsy is an invasive procedure, and tumor position substantially affects tissue sampling. The quality of the resected tissue may be poor and introduce error into the experimental predictions (Constâncio et al., 2020). In contrast, liquid biopsy can determine the methylation status even before the onset of carcinogenesis and facilitate early cancer screening. Hence, the current trend is to use liquid biopsy for DNA methylation analysis. Here, we used an additional cfDNA methylation profile of 22 cirrhotic patients from the GEO database to observe the methylation performance (Hlady et al., 2019). Among the KEGG pathway associated with the eight common biomarkers, hsa05202 (Transcriptional misregulation in cancer) contains 116 genes, of which 26 genes show |∆β| values >0.1 and adjusted p-value <0.05 in cfDNA methylation profiles. Among the 26 genes, AFF4 facilitates the expression of RUNX2 and one of the eight common biomarkers identified, HOXA9. Furthermore, Veiga et al. indicated that PBX1 is associated with cancer cell proliferation and metastasis, and it also plays an important role in the development of several cancer types, including esophageal and lung cancer (Veiga et al., 2021), which are among our selected cancer types. Several studies have shown that ARNT2 is involved in the carcinogenesis of certain cancer types, such as non-small cell lung cancer, hepatocellular carcinoma, and glioblastoma (Yang et al., 2015; Li et al., 2015; Bogeas et al., 2018). Cheng et al. revealed that CEBPB is functionally related to Menin and can be considered a therapeutic target for pancreatic cancer (Cheng et al., 2019). Additionally, Zhu et al. indicated that CEBPB could serve as a prognostic risk gene for lung cancer (Zhu et al., 2024). These observations show that the genes on the KEGG pathway associated with the eight common biomarkers, as well as the significantly differentially methylated biomarkers in cfDNA methylation profiles, also have strong effects on several cancer types.

## 5 Conclusion

DNA methylation profile analysis is one of the most promising and effective diagnostic methods for early cancer diagnosis and treatment. One of its advantages is the ability to detect the possibility of having cancer before tumor developed. This study presents an innovative approach by integrating DNA methylation profiling and comorbidity pattern analysis. Our approach can enhance the identification of biomarkers with high diagnostic potential for low-survival-rate cancers types. Eventually, we have identified eight common biomarkers (ALX3, HOXA9, HOXD8, HRH1, IRX1, NPTX2, PTPRN2, and TRIM58) and applied a hierarchical clustering method to cluster them into three functional groups based on their GO term annotations. Only one biomarker was selected from each functional group, and the combination of ALX3, NPTX2 and TRIM58 achieved the highest average prediction accuracy of 93.3% for the five initially selected cancers (brain, esophageal, liver, lung, and pancreatic cancers) and the additionally selected five common cancers (breast, colorectal, prostate, bladder, and stomach cancers).

## Data Availability

Publicly available datasets were analyzed in this study. This data can be found here: https://portal.gdc.cancer.gov/legacy-archive/search/f, https://www.ncbi.nlm.nih.gov/geo/query/acc.cgi?acc=GSE123678, https://www.ncbi.nlm.nih.gov/geo/query/acc.cgi?acc=GSE178212, https://www.ncbi.nlm.nih.gov/geo/query/acc.cgi?acc=GSE66836, https://www.ncbi.nlm.nih.gov/geo/query/acc.cgi?acc=GSE49149, accessed on 19 February 2022.
