# Supplementary material for: DNA methylation biomarker analysis from low-survival-rate cancers based on genetic functional approaches
Source: Front Bioinform. 2025 Jan 28;5:1523524. doi: 10.3389/fbinf.2025.1523524 (PMC11810926; doi:10.3389/fbinf.2025.1523524)
Supplement: Supplementary file 2 [file DataSheet1.pdf]

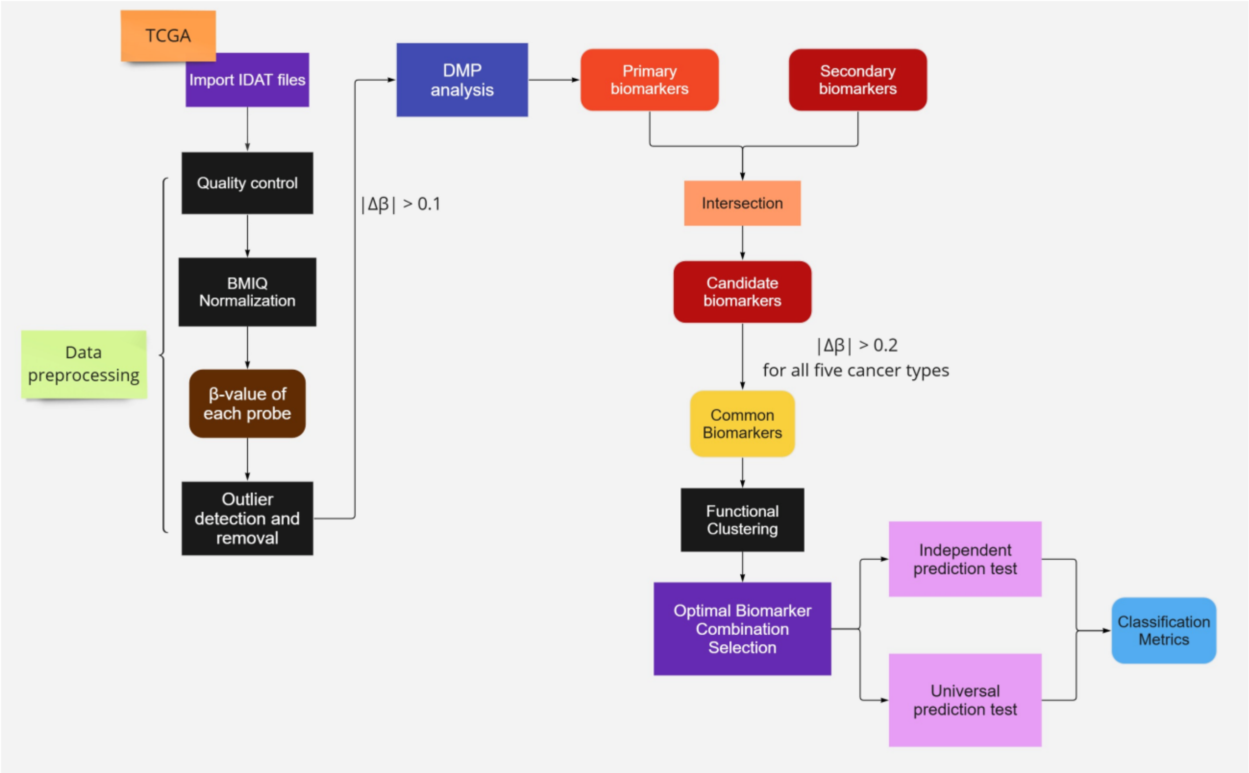

Supplementary Figure 1. The flowchart of finding common biomarkers of different target cancers

1. Import IDAT files: Download the methylation profiles from TCGA/GEO (with IDAT format) and import them through ChAMP.
2. Quality control: Remove the probes that are unsuitable for analysis.
3. BMIQ normalization: Correct the scale differences introduced by the probe design (Type I and Type II).
4. Outlier detection and removal: Apply interquartile range method on β-value and Δβ to remove outliers for each probe.
5. DMP analysis: Find the probes with significantly methylated differences.
6. Obtain the candidate biomarkers by intersecting the primary biomarkers and the secondary biomarkers:
    a. **Primary biomarkers:** Genes that contain at least one probe with |Δβ| greater than a specified thresholding value and p-value less than 0.05.
    b. **Secondary biomarkers:** Genes associated with the comorbidities of a specific cancer type.

The above sequential steps should be applied to each cancer type. Therefore, a total of five candidate biomarker sets could be discovered (one for each target cancer).

7. Common biomarkers: Select the genes that have at least one probe with an |Δβ| greater than 0.2 for each cancer type.
8. Functional Clustering
9. Optimal biomarker combination selection
    a. **Independent prediction test:** Apply SVM to independently train and test for each of the selected low-survival-rate cancers.
    b. **Universal prediction test:** Combine the subjects from the five selected low-survival-rate cancers and use SVM to train a universal prediction model. The model is then evaluated on five additional selected cancers (breast, colorectal, prostate, bladder, and stomach) to validate the classification performance.
